# Gut Microbiota-Derived Indole-3-Carboxylate Influences Mucosal Integrity and Immunity Through the Activation of the Aryl Hydrocarbon Receptors and Nutrient Transporters in Broiler Chickens Challenged With *Eimeria maxima*

**DOI:** 10.3389/fimmu.2022.867754

**Published:** 2022-06-23

**Authors:** Inkyung Park, Hyoyoun Nam, Doyun Goo, Samiru S. Wickramasuriya, Noah Zimmerman, Alexandra H. Smith, Thomas G. Rehberger, Hyun S. Lillehoj

**Affiliations:** ^1^ Animal Bioscience and Biotechnology Laboratory, Beltsville Agricultural Research Center, Agricultural Research Service, United States Department of Agriculture, Beltsville, MD, United States; ^2^ Arm & Hammer Animal and Food Production, Waukesha, WI, United States

**Keywords:** broiler chickens, *Eimeria maxima*, indole-3-carboxylate, intestinal immunity, gut health, microbial metabolites, mucosal integrity

## Abstract

Two studies were conducted to evaluate the effects of indole-3-carboxylate (ICOOH) as a postbiotic on maintaining intestinal homeostasis against avian coccidiosis. In the first study, an *in vitro* culture system was used to investigate the effects of ICOOH on the proinflammatory cytokine response of chicken macrophage cells (CMCs), gut integrity of chicken intestinal epithelial cells (IECs), differentiation of quail muscle cells (QMCs), and primary chicken embryonic muscle cells (PMCs) and anti-parasitic effect against *Eimeria maxima*. Cells to be tested were seeded in the 24-well plates and treated with ICOOH at concentrations of 0.1, 1.0, and 10.0 µg. CMCs were first stimulated by lipopolysaccharide (LPS) to induce an innate immune response, and QMCs and PMCs were treated with 0.5% and 2% fetal bovine serum, respectively, before they were treated with ICOOH. After 18 h of incubation, cells were harvested, and RT-PCR was performed to measure gene expression of proinflammatory cytokines of CMCs, tight junction (TJ) proteins of IECs, and muscle cell growth markers of QMCs and PMCs. In the second study, *in vivo* trials were carried out to study the effect of dietary ICOOH on disease parameters in broiler chickens infected with *E. maxima*. One hundred twenty male broiler chickens (0-day-old) were allocated into the following four treatment groups: 1) basal diet without infection (CON), 2) basal diet with *E. maxima* (NC), 3) ICOOH at 10.0 mg/kg feed with *E. maxima* (HI), and 4) ICOOH at 1.0 mg/kg feed with *E. maxima* (LO). Body weights (BWs) were measured on 0, 7, 14, 20, and 22 days. All groups except the CON chickens were orally infected with *E. maxima* on day 14. Jejunal samples were collected for lesion score and the transcriptomic analysis of cytokines and TJ proteins. *In vitro*, ICOOH increased the expression of TJ proteins in IECs and decreased IL-1β and IL-8 transcripts in the LPS-stimulated CMCs. *In vivo*, chickens on the HI diet showed reduced jejunal IL-1β, IFN-γ, and IL-10 expression and increased expression of genes activated by aryl hydrocarbon receptors and nutrient transporters in *E. maxima*-infected chickens. In conclusion, these results demonstrate the beneficial effects of dietary ICOOH on intestinal immune responses and barrier integrity in broiler chickens challenged with *E. maxima*. Furthermore, the present finding supports the notion to use microbial metabolites as novel feed additives to enhance resilience in animal agriculture.

## 1 Introduction

Recently, modern advances in omics technologies such as metabolomics, metagenomics, transcriptomics, and proteomics are helping to understand the mechanisms between gut microbiota and the brush border of the intestinal epithelium (1–3). Especially, gut microbiota-generating metabolites have been assessed as molecules that are detected by pattern recognition receptors (PRRs) displayed by intestinal epithelial cells (IECs) (4, 5). Recognition of these pathogen-associated molecular patterns and damage-associated molecular patterns *via* PRRs influence both the magnitude and the quality of the immune responses (6, 7). Certain metabolites such as short-chain fatty acids, bacteriocins, functional peptides, and proteins have been reported to exert beneficial effects on the host through modulating intestinal immune responses and influencing epithelial integrity (8–10). Indeed, the reduction of microbial byproducts needed for digestion processes in the intestine demonstrated a possibility to lead to indigestion and diseases through an experiment on whether intestinal metabolites affect intestinal immunity (6).

In our previous study, dietary *Bacillus subtilis* 1781 and 747 supplementation showed beneficial effects on intestinal immune responses, epithelial barrier integrity, and growth performance of chicken infected with *E. maxima* (11). In a further study aimed to understand the practical mechanisms of these beneficial results through metabolomics, the global metabolite analysis was conducted on ilea of *Bacillus*-fed chickens (12). A total of 674 metabolites were identified from the global metabolite profiling. Of these, the levels of 209 metabolites were increased over the control, and 461 were decreased. Eight metabolites related to indole were increased, which were indole-3-carboxylate (ICOOH), indole acetate, 2-oxindole-3-acetate, 3-formylindole, 3-indoleglyoxylic acid, methyl indole-3-acetate, 5-hydroxyindoleacetate, and indolelactate. Among these indole metabolites, ICOOH level was increased 2.35-fold compared to the control group, while that of others was less than 1.4-fold.

Indole glucosinolates, which have side chains derived from tryptophan, are abundant in *Brassica* vegetables such as broccoli, cabbage, cauliflower, mustard greens, and rutabagas (13, 14), and indole compounds have been reported to exhibit anti-inflammatory and anti-carcinogenic properties (15, 16). In addition to indole compounds in plants, gut microbiota forms various indole derivatives from tryptophan catabolism, which have been shown to influence the modulation of immune responses through aryl hydrocarbon receptor (AhR) signaling and regulation of barrier function through the nutrient transporters and pregnane X receptor (PXR) (17, 18). However, there are very few reports on the intestinal biological function of ICOOH even though other similar indole compounds are well known to manipulate host immune responses locally or systemically (19–22).

We hypothesized that ICOOH could be a good health-promoting postbiotic. Therefore, the objectives of the present study were as follows: 1) *in vitro* evaluation of the effects of ICOOH on the host innate immune response using chicken macrophage cells (CMCs), on the barrier integrity of chicken IECs, on anticoccidial ability against *Eimeria maxima* sporozoites, and on myogenic differentiation of quail muscle cells (QMCs) and primary chicken embryonic muscle cells (PMCs); and 2) the *in vivo* characterization of the dietary ICOOH on growth performance, intestinal immunity, epithelial integrity, and nutrient transporters in young broiler chickens challenged with *E. maxima*. These studies will provide information on mechanisms by which the alteration of intestinal metabolites generated by dietary direct-fed microbes exerts beneficial physiological changes on the host.

## 2 Materials and Methods

### 2.1 Experiment 1: *In Vitro* Study

#### 2.1.1 Culture of Chicken Intestinal Epithelial Cells and Chicken Macrophage Cells

IECs (2 × 10^5^/ml, 8E11; Micromol, Karlsruhe, Germany) and CMCs (2 × 10^5^/ml, HD11; Micromol) were seeded in 24-well plates and maintained in the Dulbecco’s modified Eagle medium (DMEM)/F-12 (HyClone, Logan, UT, USA) supplemented with 10% heat-inactivated fetal bovine serum (FBS; HyClone) and 1% penicillin (10,000 unit/ml)/streptomycin (10 mg/ml, Gibco, Grand Island, NY, USA). Both cell types were incubated at 41°C in a humidified atmosphere with 5% CO_2_ for 24 h for cell adhesion. After 24 h, lipopolysaccharide (LPS; Sigma-Aldrich, St. Louis, MO, USA; L2630) at a concentration of 1.0 µg/ml and ICOOH (Sigma-Aldrich, 395307) at concentrations of 0.0, 0.1, 1.0, and 10.0 µg/ml were administrated to each well in the 24-well plates. After 18 h, lysis buffer (Qiagen, Valencia, CA, USA) and 2-mercaptoethanol (Sigma-Aldrich) were used to harvest all cells. RNA was isolated from the IECs and the CMCs using the RNeasy Isolation Kit (Qiagen) in a QIAcube (Qiagen) for performing quantitative real-time PCR (qRT-PCR) analysis. All experiments were replicated independently three times.

#### 2.1.2 Anticoccidial Assay Against *Eimeria maxima*

The direct effect of ICOOH on *Eimeria* parasites was tested using *in vitro* sporozoite killing assay as described (23). Briefly, fresh sporulated oocysts were disrupted with 0.5-mm glass beads for 10 s using the Mini-Bead beater (BioSpec Products, Bartlesville, OK, USA). The released sporocysts were washed in chilled Hanks’ balanced salt solution (HyClone) and treated with excystation media (0.25% trypsin and 0.014 M of taurocholic acid, pH 7.4) at 41°C for 1 h to release sporozoites. Sporozoites (2.5 × 10^5^) were seeded in each well of a 96-well plate. Chicken NK-lysins (Genscript, Piscataway, NJ, USA) at concentrations of 1.0, 10, and 100 µg/ml were used as positive controls. Three different ICOOH doses, low (0.1 µg/ml), medium (1.0 µg/ml), and high (10 µg/ml), were used to treat freshly prepared live sporozoites and then incubated at 41°C for 3 h. Fluorescent dye (AO/PI staining solution, Nexcelom Bioscience LLC, Lawrence, MA, USA) was administrated to each well in a 1:1 ratio, and live sporozoites were counted using Cellometer (Nexcelom Bioscience). All experiments were replicated more than three times independently.

#### 2.1.3 Quail Muscle Cell Culture

QMCs (2 × 10^5^/ml) were seeded in 24-well plates as per the methods described previously (24). The QMCs were maintained in Medium 199 (HyClone) containing 10% FBS and 1% penicillin/streptomycin until cells reached 70% confluence. Media in 12 wells were replaced by Medium 199 containing 0.5% FBS with 1% penicillin/streptomycin to induce cell differentiation, and in the remaining 12 wells of the same plate, media were replaced by a basic Medium 199 containing 10% FBS to maintain cell proliferation. Four different ICOOH doses (0.0, 0.1, 1.0, and 10.0 µg/ml) were administrated to each well in the 24-well plates. After incubation at 41°C in a humidified atmosphere with 5% CO_2_ for 18 h, all cells were collected in lysis buffer and 2-mercaptoethanol. RNA from QMCs was isolated using the RNeasy Isolation Kit in the QIAcube for performing qRT-PCR analysis. All experiments were replicated more than three times independently.

#### 2.1.4 Primary Chicken Embryonic Muscle Cell Culture

Eggs for the embryonic muscle cell culture were obtained from Moyer’s hatchery (Quakertown, PA, USA). The PMC culture was modified based on the method described by Hassan et al. (25). Briefly, eggs were incubated at 41°C and 80% humidity. The pectoralis major region of the embryos was extracted at 13 days; it was minced and digested with 0.05% trypsin-EDTA (Sigma-Aldrich) at 37°C for 20 min. The PMCs were washed 2–3 times with Hanks’ balanced salt solution (Sigma-Aldrich) and seeded (2 × 10^5^/ml) in 24-well plates. The PMCs were maintained in DMEM (HyClone) containing 10% FBS and 1% penicillin/streptomycin until visual confirmation of 70% confluence was attained. Culture media in 12 wells were replaced as DMEM containing 2% FBS with 1% penicillin/streptomycin to induce cell differentiation, and in the remaining 12 wells of the same plate, media were replaced by basic DMEM containing 10% FBS to maintain cell proliferation. ICOOH at concentrations of 0.0, 0.1, 1.0, and 10.0 µg/ml was added to each well in the 24-well plates. After incubation at 41°C in a humidified atmosphere with 5% CO_2_ for 18 h, all cells were collected in lysis buffer and 2-mercaptoethanol. RNA from the PMCs was extracted using the RNeasy Isolation Kit in the QIAcube or performing qRT-PCR analysis. RNA was eluted in 30 μl of RNase-free water. All experiments were replicated independently more than three times.

#### 2.1.5 Reverse Transcription From *In Vitro* Samples

The quantity of RNA was assessed using the NanoDrop (ND-1000) spectrophotometer (NanoDrop Products, Wilmington, DE, USA) according to the absorbance at 260 nm. RNA purity was evaluated based on the OD260/OD280 ratio. Total RNA (1 µg) was then reverse-transcribed to cDNA using the QuantiTect^®^ reverse transcription (RT) kit (Qiagen). Briefly, the RNA sample was incubated with genomic DNA wipeout buffer (Qiagen) at 42°C for 2 min to remove any genomic DNA contamination.

RT of the genomic DNA-depleted sample was performed by the addition of the Quantiscript Reverse Transcriptase, Quantiscript RT buffer, and RT primer mix. The reaction was performed in a thermal cycler (Mastercycler^®^ EP Gradient S; Eppendorf, Hauppauge, NY, USA). The cycling conditions were 42°C for 30 min, followed by reverse transcriptase inactivation at 95°C for 3 min. The cDNA samples were divided into aliquots and stored at −20°C.

#### 2.1.6 Analysis of Cytokines, Tight Junction Proteins, and Markers of Muscle Cell Growth by qRT-PCR

Proinflammatory cytokines (IL-1β, IL-6, and IL-8) levels were measured in IECs and CMCs using extracted RNA samples. For analysis of tight junction (TJ) proteins (occludin, ZO-1, and MUC-2), qRT-PCR was conducted using RNA samples extracted from IECs. Proliferation and differentiation markers of muscle cells, Pax7 and MyoG, were determined using RNA samples obtained from QMCs and PMCs. qRT-PCR was performed using the Agilent Mx3000 P QPCR System (Agilent Technologies, Santa Clara, CA, USA) and the Brilliant SYBR Green qRT-PCR Master Mix (StrataGene, La Jolla, CA, USA). Oligonucleotide primer sequences and product size used for qRT-PCR are listed in **Table 1**. A melting curve was obtained at the end of each run to verify the presence of a single amplification product without primer dimers. Standard curves were generated using serial, 5-fold dilutions of cDNA. The fold changes in each transcript were normalized to glyceraldehyde-3-phosphate dehydrogenase and are relative to the transcript expression in the unstimulated control group (normalized to 1) using the comparative ΔΔCt method as previously described (26).

**Table 1:** Ingredient composition of basal diet (as-fed basis, %, unless otherwise indicated).

Ingredients (%)	Basal diet
Corn	55.78
Soybean meal	37.03
Soybean oil	2.97
Dicalcium phosphate	1.80
Calcium carbonate	1.51
Salt	0.38
Poultry Vit Mix^1^	0.22
Poultry Mineral Mix^2^	0.15
dl-Methionine	0.10
Choline-chloride, 60%	0.06
Total	100.00
Calculated values (%)	
CP, %	24.00
Ca, %	1.20
AP, %	0.51
Lys, %	1.40
Met, %	0.49
Cys + Met, %	0.80
ME, Mcal/kg	3.5

CP, crude protein; AP, available phosphorus.

^1^ Vitamin mixture provided the following nutrients per kg of diet: vitamin A, 2,000 IU; vitamin D3, 22 IU; vitamin E, 16 mg; vitamin K, 0.1 mg; vitamin B1, 3.4 mg; vitamin B2, 1.8 mg; vitamin B6, 6.4 mg; vitamin B12, 0.013 mg; biotin, 0.17 mg; pantothenic acid, 8.7 mg; folic acid, 0.8 mg; niacin, 23.8 mg.

^2^ Mineral mixture provided the following nutrients per kg of diet: Fe, 400 mg; Zn, 220 mg; Mn, 180 mg; Co, 1.3 mg; Cu, 21 mg; Se, 0.2 mg.

### 2.2 Experiment 2: *In Vivo* Study

**Figure 1:**
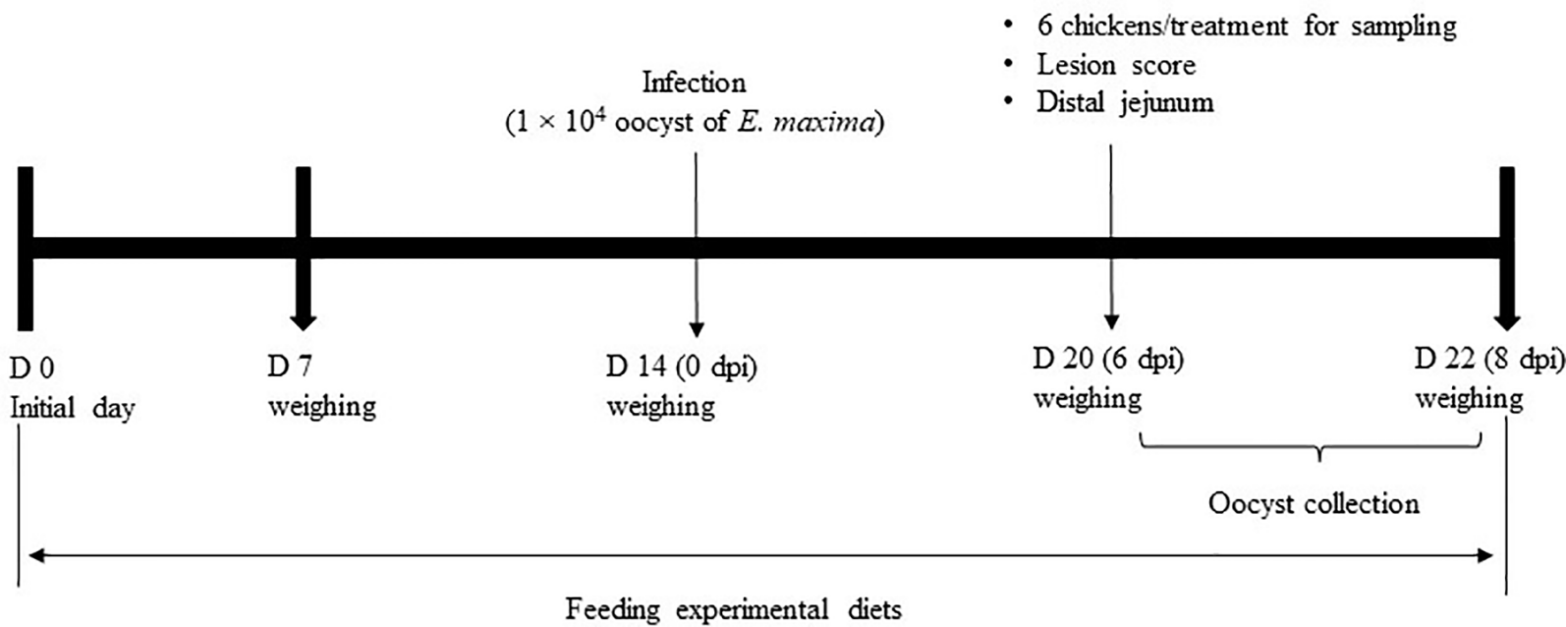
Schematic outline of the experimental design in experiment 2.

This animal experiment was approved by the Beltsville Agricultural Research Center Institutional Animal Care and Use Committee (#19-018). **Figure 1** depicts the schematic outline of the experimental design used for the execution of the study.

#### 2.2.1 Chickens and Experimental Design

A total of 120 newly hatched (Ross 708) male broiler chickens at 0 days of age were purchased from Longenecker’s hatchery (Elizabethtown, PA, USA). The day after the chickens arrived at the Beltsville ARS facility, they were weighed to perform adjustments to obtain the same body weight (BW) per treatment and allocated to four dietary treatments in a randomized complete block design. The dietary treatments included the provision of a corn- and soybean meal-based (basal) diet without infection (CON, **Table 2**), a basal diet with *E. maxima* infection (NC), ICOOH at 10.0 mg/kg feed (HI), and 1.0 mg/kg feed (LO). Crystalline ICOOH was purchased from Sigma-Aldrich. Each treatment group was allocated to six wire-bottom cages with five chickens per cage (0.65 × 0.75 m^2^). The chickens were provided with *ad libitum* access to water and feed throughout the experimental period. A schematic outline of the experimental design is shown in **Figure 1**.

**Table 2:** Oligonucleotide primer sequences for qRT-PCR.

Type	Target gene	Primer sequence (5′–3′)	PCR product size (kb)
Reference	GAPDH	F-GGTGGTGCTAAGCGTGTTAT	264
		R-ACCTCTGCCATCTCTCCACA	
Proinflammatory	IL-1β	F-TGGGCATCAAGGGCTACA	244
		R-TCGGGTTGGTTGGTGATG	
	IL-6	F-CAAGGTGACGGAGGAGGAC	254
		R-TGGCGAGGAGGGATTTCT	
	IL-8	F-GGCTTGCTAGGGGAAATGA	200
		R-AGCTGACTCTGACTAGGAAACTGT	
	TNFSF15	F-CCTGAGTATTCCAGCAACGCA	292
		R-ATCCACCAGCTTGATGTCACTAAC	
Th	IFN-γ	F-AGCTGACGGTGGACCTATTATT	259
		R-GGCTTTGCGCTGGATTC	
	IL-10	F-CGGGAGCTGAGGGTGAA	272
		R-GTGAAGAAGCGGTGACAGC	
TJ proteins	Claudin-1	F-CCTGATCACCCTCTTGGGAG	145
		R-GCTGCACTCACTCATTGGCT	
	Claudin-2	F-CCTGCTCACCCTCATTGGAG	145
		R-GCTGAACTCACTCTTGGGCT	
	JAM-2	F-AGCCTCAAATGGGATTGGATT	59
		R-CATCAACTTGCATTCGCTTCA	
	Occludin	F-GAGCCCAGACTACCAAAGCAA	68
		R-GCTTGATGTGGAAGAGCTTGTTG	
	ZO-1	F-CCGCAGTCGTTCACGATCT	63
		R-GGAGAATGTCTGGAATGGTCTGA	
	ZO-2	F-ATCCAAGAAGGCACCTCAGC	100
		R-CATCCTCCCGAACAATGC	
AhR	CYP1A4	F-CCGTGACAACCGCCCTGTCC	359
		R-GAGTTCGGTGCCGGCTGCAT	
	CYP1A5	F-GGACCGTTGCGTGTTTAT	469
		R-CTCCCACTTGCCTATGTTTT	
Muscle cell	MyoG	F-TGACCCTGTGCCCTGAAAGC	178
		R-TCGTTCACCTTCTTCAGCCTCC	
	Pax7	F-AAGGCCAAGCACAGCATAGA	108
		R-GCGCTGCTTCCTCTTCAAAG	
Nutrient transports	B^0^AT	F-GGGTTTTGTGTTGGCTTAGGAA	60
		R-TCCATGGCTCTGGCAGAGAT	
	B^0+^AT	F-CAGTAGTGAATTCTCTGAGTGTGAAGCT	88
		R-GCAATGATTGCCACAACTACCA	
	CAT1	F-CCAAGCACGCTGATAAAG	75
		R-TACTCACAATAGGAAGAAGGG	
	EAAT	F-TGCTGCTTTGGATTCCAGTGT	79
		R-AGCAATGACTGTAGTGCAGAAGTAATATATG	
	GLUT1	F-CTTTGTCAACCGCTTTGG	65
		R-TGTGCCCCGGAGCTTCT	
	GLUT2	F-TCATTGTAGCTGAGCTGTT	68
		R-CGAAGACAACGAACACATAC	
	GLUT5	F-TTGCTGGCTTTGGGTTGTG	60
		R-GGAGGTTGAGGGCCAAAGTC	
	LAT1	F-GATTGCAACGGGTGATGTGA	70
		R-CCCCACACCCACTTTTGTTT	
	LAT2	F-TCAGCTTCAGTTACTGGTT	68
		R-GCACAACCACGAGAAATAC	
	SGLT	F-GCCGTGGCCAGGGCTTA	71
		R-CAATAACCTGATCTGTGCACCAGT	

Th, T helper cells; TJ, tight junction; AhR, aryl hydrocarbon receptor; B^0^AT, Na+-dependent amino acid transporter; B^0+^AT, Na+-independent amino acid transporter; CAT1, cationic amino acid transporter; CYP1A4, chicken cytochrome P-450 enzyme; CYP1A5, chicken cytochrome P-450 enzyme; EAAT, excitatory amino acid transporter; GLUT1, glucose transporter 1; GLUT2, glucose transporter 2; GLUT5, glucose transporter 5; LAT1, L-type amino acid transporter 1; LAT2, Na^+^-dependent neutral/cationic amino acid transporter; MyoG, Myogenin; Pax7, Paired Box 7; SGLT, sodium-glucose transporter.

#### 2.2.2 Determination of Body Weight

The BW of chickens was measured on days 0, 7, 14, 20, and 22 to calculate average daily gain (ADG). Dead chickens were removed and weighed to perform adjustments for the growth data.

#### 2.2.3 Oral Infection With *Eimeria maxima*

All chickens, except for those in the CON group, were infected by oral gavage at day 21 with *E. maxima* (1.0 × 10^4^ oocysts/chicken, Beltsville strain 41A) as per previously described methods (11). The purity of the infected *E. maxima* was confirmed by conducting a DNA genotyping test (27).

#### 2.2.4 Collection of Intestinal Samples

One chicken with an average BW from each cage was euthanized by cervical dislocation on day 20, and its intestines were removed for further analysis. From each intestine, a small section (2 cm) of the distal jejunum without contents was collected aseptically and stored in RNAlater^®^ (Invitrogen, Carlsbad, CA, USA) at −20°C until subsequent analysis.

#### 2.2.5 Jejunal Lesion Scoring

With the use of the 15-cm long distal jejunum sample, gut lesion scoring was performed on day 20. Lesions were scored on a scale from 0 (none) to 4 (high) by four independent observers in a blinded manner, as per methods described previously (28).

#### 2.2.6 Fecal Oocyst Shedding

From days 20 to 22 (6 to 8 days post-infection (dpi)), fecal samples were collected, and the number of oocysts was counted as per protocols described previously (27) using the McMaster chamber according to the following formula:

total oocysts/chicken = [oocyst count × dilution factor × (fecal sample volume/counting chamber volume)]/number of chickens per cage.

#### 2.2.7 Isolation of RNA and Reverse Transcription From Jejunal Samples

Total RNA was isolated from jejunum samples that were stored in RNAlater^®^ according to the manufacturer’s recommendations. Approximately 50 mg of the jejunal tissue was homogenized in 1 ml of TRIzol (Invitrogen) using a handheld homogenizer (TissueRuptor; Qiagen). Chloroform was added to the homogenized sample. The samples were centrifuged at 12,000 × *g* for 15 min at 4°C for phase separation. RNA present in the colorless upper aqueous phase was then precipitated using 100% isopropanol (Sigma-Aldrich). The RNA pellet was washed with 75% ethanol (Sigma-Aldrich), air-dried, and re-suspended in RNase-free water (Invitrogen). The quantity of RNA was assessed using the NanoDrop (ND-1000) spectrophotometer (NanoDrop Products, Wilmington, DE, USA) according to the absorbance at 260 nm. RNA purity was evaluated based on the OD260/OD280 ratio. Total RNA (1 µg) was then reverse-transcribed to cDNA using the QuantiTect^®^ RT kit (Qiagen). Briefly, the RNA sample was incubated with genomic DNA wipeout buffer at 42°C for 2 min to remove any genomic DNA contamination. RT of the genomic DNA-depleted sample was performed by the addition of the Quantiscript Reverse Transcriptase, Quantiscript RT buffer, and RT primer mix. The reaction was performed in a thermal cycler (Mastercycler^®^ EP Gradient S; Eppendorf, Hauppauge, NY, USA). The cycling conditions were 42°C for 30 min, followed by reverse transcriptase inactivation at 95°C for 3 min. The cDNA samples were divided into aliquots and stored at −20°C.

#### 2.2.8 Gene Expression Analysis by qRT-PCR From the Extracted RNA

The oligonucleotide primer sequences used for qRT-PCR are listed in **Table 1**. The expression of various cytokines and intestinal TJ proteins was evaluated in the jejunum, including cytokines (IL-1β, IL-6, IL-10, IL-17F, IFN-γ, and TNFSF15), TJ proteins (claudin-1, claudin-2, JAM-2, occludin, ZO-1, and ZO-2), and nutrient transporters (B^0^AT, B^0+^AT, CAT1, EAAT, GLUT1, GLUT2, GLUT5, LAT1 LAT2, and SGLT). Glyceraldehyde-3-phosphate dehydrogenase was used as the reference gene. Amplification and detection were performed using the StrataGene Mx3000P qPCR system (Agilent Technologies Inc.) and RT^2^ SYBR Green qPCR master mix (Qiagen). Each sample was analyzed in triplicate, and non-specific primer amplification was assessed *via* the inclusion of no-template controls. Standard curves were generated using log10 diluted standards of RNA, and the levels of individual transcripts were normalized to those of glyceraldehyde-3-phosphate dehydrogenase in the Q-gene program (23).

#### 2.2.9 Statistical Analysis

*In vitro* data for each response were analyzed using the Proc GLM in SAS version 9.4 (SAS Inc., Cary, NC, USA). All data were accepted when skewness and kurtosis ranged in values at ±3 and ±7, respectively. *In vivo* data were analyzed using a mixed model (PROC MIXED) in SAS. Each cage was considered an experimental unit. The results are shown as least squares mean values and pooled SEM. Probability values of less than 0.05 were considered significantly different. In cases where the overall effect was significant, the mean values were compared in a pairwise manner (PDIFF option).

## 3 Results

### 3.1 Experiment 1: *In Vitro* Studies

#### 3.1.1 Effect of ICOOH on the Gene Expression of Biomarkers Associated With Gut Integrity

**Figure 2:**
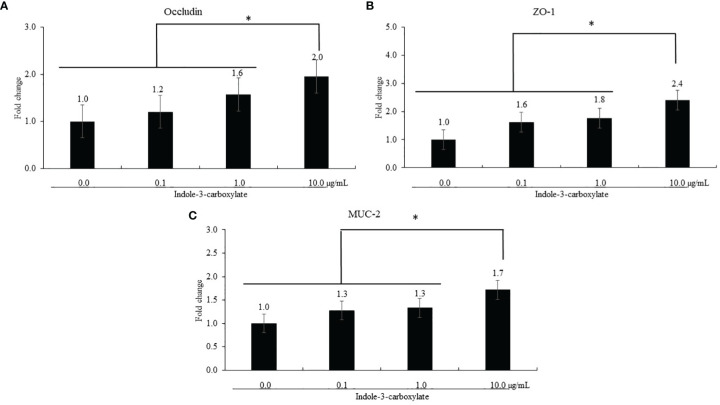
Alteration of tight junction proteins and mucin in chicken epithelial cells (IECs) by indole-3-carboxylate. Each bar represents the mean ± SEM (n = 3). Transcript levels of the tight junction proteins were measured using quantitative RT-PCR and normalized to GAPDH transcript levels. * *p* < 0.05. ICOOH at 10.0 μg/ml increased (*p* < 0.05) gene expression levels of **(A)** occludin, **(B)** ZO-1, and **(C)** MUC-2 more than 1.7 times compared to that of ICOOH at 0.0 μg/ml (control group).

ICOOH at a dose of 10.0 µg/ml increased occludin (*p* = 0.041, 1.0- to 2.0-fold), ZO-1 (*p* < 0.001, 1.0- to 2.4-fold), and MUC-2 (*p* < 0.001, 1.0- to 1.7-fold) gene expression compared to that in each control group (**Figure 2**). However, ICOOH at doses of 0.1 and 1.0 µg/ml did not change (*p* > 0.05) these TJ proteins and mucin levels compared to ICOOH at 0.0 µg/ml (control group).

#### 3.1.2 Effect of ICOOH on Proinflammatory Responses of Chicken Macrophage Cells

**Figure 3:**
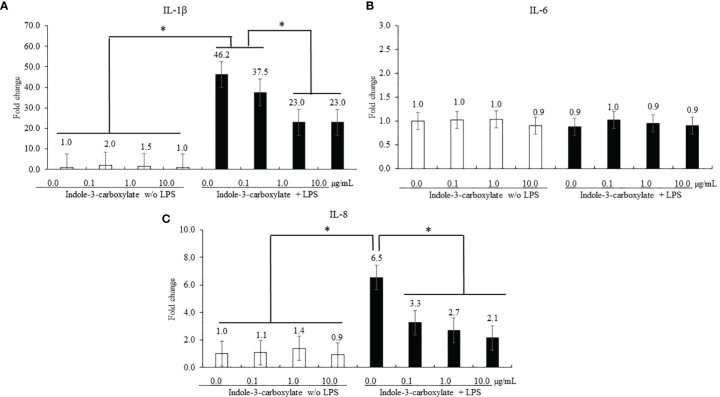
Secretion of proinflammatory cytokines in chicken macrophage cells (CMCs) by lipopolysaccharide (LPS) and indole-3-carboxylate. Each bar represents the mean ± SEM (n = 3). Transcript levels of the cytokines were measured using quantitative RT-PCR and normalized to GAPDH transcript levels. Significant results are marked as *p < 0.05. LPS, lipopolysaccharide. ICOOH at 1.0 and 10.0 μg/ml suppressed gene expression levels of **(A)** IL-1β and **(C)** IL-8, which were increased by LPS.

Regardless of ICOOH administration, LPS stimulation of CMCs increased (*p* < 0.001) the average IL-1β level (1.4- to 32-fold) in CMCs compared to that in the groups without LPS treatment, whereas ICOOH at doses of 1.0 (46.2- to 23.0-fold) and 10.0 µg/ml (46.2- and 23.0-fold) decreased (*p* < 0.001) IL-1β levels compared to that of control in LPS treatment group (**Figure 3A**). However, IL-6 levels in CMCs were not changed (*p* > 0.05) by either LPS or ICOOH administration (**Figure 3B**). LPS stimulation regardless of ICOOH administration increased (*p* = 0.038) the average IL-8 levels (1.1- to 3.7-fold) compared to that of groups without LPS stimulation. Among the LPS groups, ICOOH regardless of doses decreased (*p* < 0.008) IL-8 levels (6.5- to 2.7-fold) compared to those in control with LPS administration without ICOOH (**Figure 3C**).

#### 3.1.3 Anticoccidial Activity of ICOOH Against Sporozoites of *Eimeria maxima*

**Figure 4:**
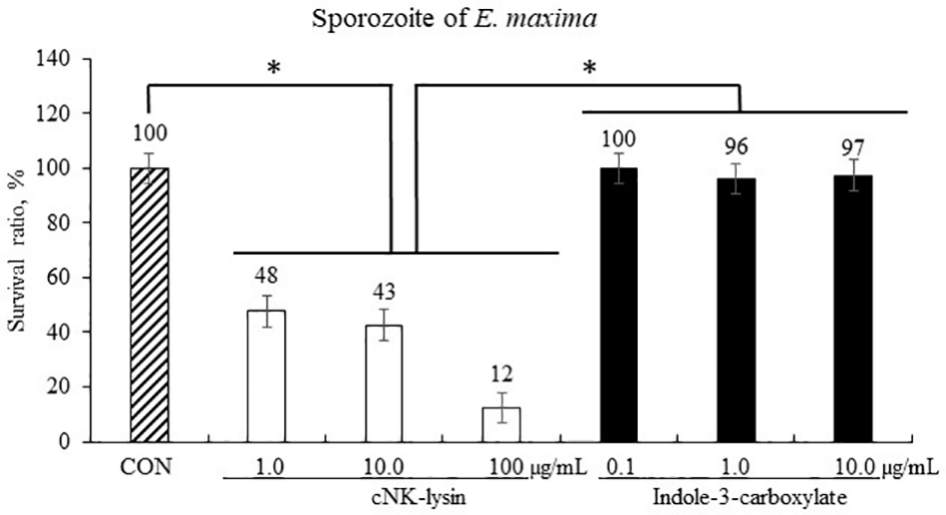
Anticoccidial effect of indole-3-carboxylate on sporozoites of *Eimeria maxima* in experiment 1. Each bar represents the mean ± SEM (n = 3). Significant results are marked as *(*p* < 0.05). CON = 2.5 × 10^5^ sporozoites/ml. .

Host-derived cNK-lysin decreased (*p* < 0.001) the survival rate of sporozoites of *E. maxima* in a dose-dependent manner by 12% to 48% compared to that in the CON group (**Figure 4**). Indole-3-carboxylate did not change (*p* > 0.05) the survival ratio of sporozoites of *E. maxima* compared to that in the CON group.

#### 3.1.4 Effect of ICOOH on the Proliferation and Differentiation of Quail Muscle Cells and Primary Chicken Embryonic Muscle Cells

**Figure 5:**
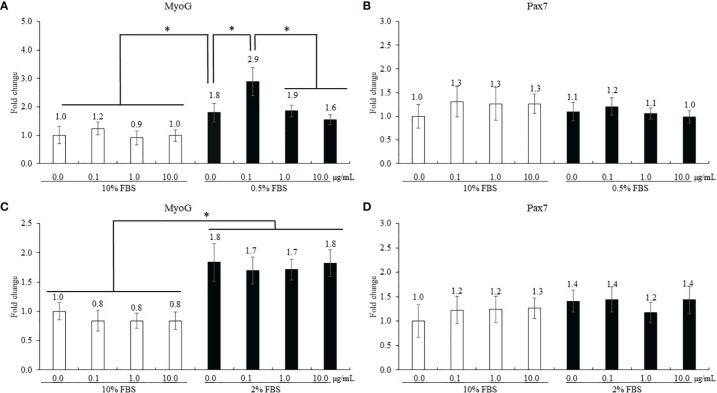
Proliferation and differentiation of quail muscle cells (QMCs; **A** and **B**) and primary chicken embryonic muscle cells (PMCs; **C** and **D**) by fetal bovine serum (FBS) concentration and indole-3-carboxylate. Each bar represents the mean ± SEM (n = 3). Transcript levels of the MyoG and Pax7 were measured using quantitative RT-PCR and normalized to GAPDH transcript levels. Significant results are marked as follows: *p < 0.05.

The treatment using 0.5% FBS without ICOOH addition increased (*p* = 0.046, 1.0- to 1.8-fold) MyoG levels of QMCs compared to that of control using 10% FBS (**Figure 5A**). However, ICOOH at 0.1 µg/ml further increased (*p* = 0.039, 1.8- to 2.9-fold) MyoG levels of QMCs using 0.5% FBS compared to other doses of ICOOH in 0.5% FBS groups. Pax7 levels (**Figure 5B**) of QMCs were not changed by FBS or ICOOH concentrations. In PMCs, regardless of ICOOH concentration, 2% FBS increased (*p* < 0.007) average MyoG levels (0.9- to 1.8-fold) compared to the average of groups in 10% FBS (**Figure 5C**). However, ICOOH administration did not affect (*p* > 0.05) MyoG levels in both 10% and 2% FBS groups. Pax7 levels of PMCs did not change (*p* > 0.05) with respect to the ICOOH dose or FBS concentration used (**Figure 5D**).

### 3.2 Experiment 2: *In Vivo* Studies

#### 3.2.1 Proinflammatory and Th Cytokines

**Figure 6:**
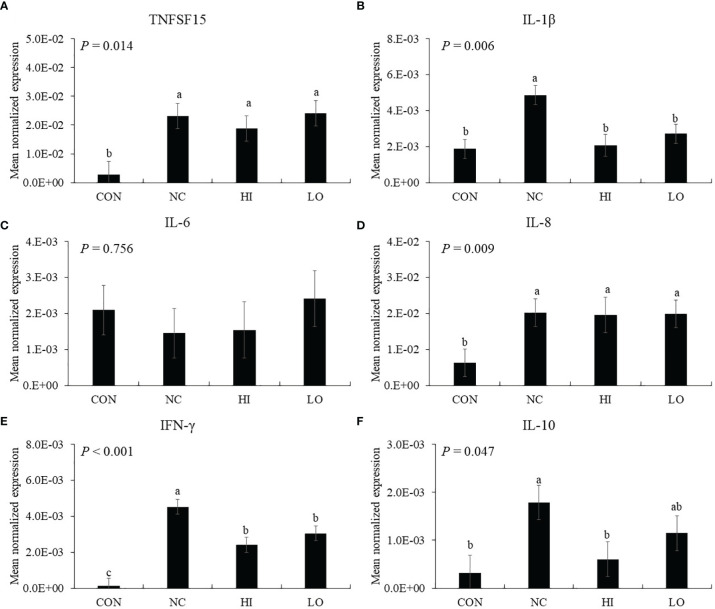
Transcripts of proinflammatory **(A–D)** and Th **(E** and **F)** cytokines in jejunum of chickens fed diet supplemented with indole-3-carboxylate during infection with *Eimeria maxima* in experiment 2. CON, basal diet; NC, basal diet for infected chickens; HI, indole-3-carboxylate at 10.0 mg/kg feed; LO, indole-3-carboxylate at 1.0 mg/kg feed. All chickens, except for CON, were infected by oral gavage at day 14 with 1.0 × 10^4^ oocysts/chicken of *E. maxima*. ^a~c^ Bars with no common letter differ significantly (*p* < 0.05). Each bar represents the mean ± SEM (n = 6). The data were collected on day 20 (6 days post-infection). Transcript levels of the cytokines were measured using quantitative RT-PCR and normalized to GAPDH transcript levels.

Infection (NC group) with *E. maxima* increased the gene expression of TNFSF15 (*p* = 0.006, 2.9 × 10^−3^ to 2.3 × 10^−2^), IL-1β (*p* = 0.001, 1.9 × 10^−3^ to 4.9 × 10^−3^), and IL-8 (*p* = 0.022 6.3 × 10^−3^ to 2.0 × 10^−2^) in the distal jejunum compared to that of the CON group (**Figures 6A, B, D**). In the dietary ICOOH supplementation, the HI (*p* = 0.004, 4.9 × 10^−2^ to 2.1 × 10^−3^) and LO (*p* = 0.013, 4.9 × 10^−3^ to 2.8 × 10^−3^) groups decreased the expression of IL-1β compared to that of the NC group; however, jejunal TNFSF15 and IL-8 levels were not changed by dietary ICOOH supplementation. The IL-6 level was not also changed by *E. maxima* infection and dietary ICOOH supplementation (**Figure 6C**).

In the absence of ICOOH, challenge infection (NC group) with *E. maxima* increased the gene expression of IFN-γ (*p* < 0.001, 1.2 × 10^−4^ to 4.5 × 10^−3^) and IL-10 (*p* = 0.012, 3.2 × 10^−4^ to 1.8 × 10^−3^) in the distal jejunum compared to that of the CON group (**Figures 6E, F**). The HI (*p* = 0.003, 4.5 × 10^−3^ to 2.1 × 10^−3^) and LO (*p* = 0.023, 4.5 × 10^−3^ to 1.4 × 10^−3^) groups decreased the gene expression of IFN-γ compared to that of NC. The HI decreased (*p* = 0.036, 1.8 × 10^−3^ to 6.0 × 10^−4^) the IL-10 level compared to that of NC, but the IL-10 level of LO compared to NC and HI has no difference.

#### 3.2.2 Aryl Hydrocarbon Receptors in Jejunum

**Figure 7:**
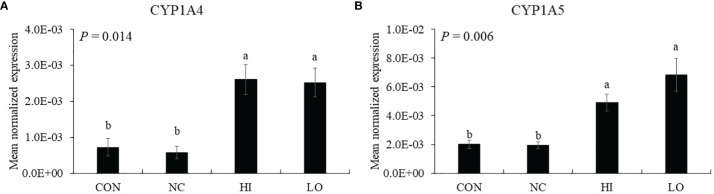
Markers of aryl hydrocarbon receptor activation in jejunum of chickens fed diet supplemented with indole-3-carboxylate during infection with *Eimeria maxima* in experiment 2. CON, basal diet; NC, basal diet for infected chickens; HI, indole-3-carboxylate at 10.0 mg/kg feed; LO, indole-3-carboxylate at 1.0 mg/kg feed. All chickens, except for CON, were infected by oral gavage at day 14 with 1.0 × 10^4^ oocysts/chicken of *E. maxima*. ^a~b^ Bars with no common letter differ significantly (*p* < 0.05). Each bar represents the mean ± SEM (n = 6). The data were collected on day 20 (6 days post-infection). Transcript levels of the CYP1A4 and CYP1A5 were measured using quantitative RT-PCR and normalized to GAPDH transcript levels.

Dietary ICOOH supplementation regardless of doses increased (*p* < 0.01) the expression of CYP1A4 (HI, 7.2 × 10^4^ to 2.6 × 10^3^; LO, 7.2 × 10^4^ to 2.5 × 10^3^) and CYP1A5 (HI, 2.0 × 10^3^ to 4.9 × 10^3^; LO, 2.0 × 10^3^ to 6.8 × 10^3^) compared to that of control (CON). Infection with *E. maxima* did not affect (*p* > 0.05) the concentration in the jejunum of chicken compared to that of CON (**Figures 7A, B**).

#### 3.2.3 Tight Junction Proteins

**Figure 8:**
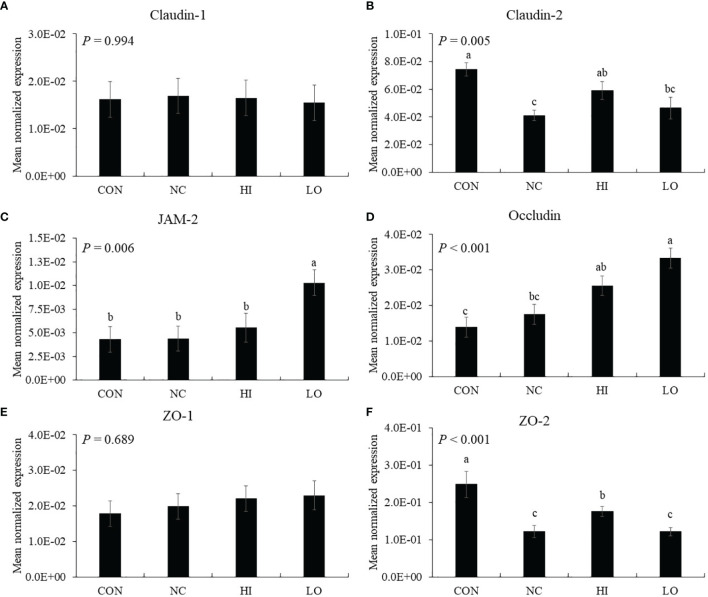
Transcripts of tight junction protein in jejunum of chickens fed diet supplemented with indole-3-carboxylate during infection with *Eimeria maxima* in experiment 2. CON, basal diet; NC, basal diet for infected chickens; HI, indole-3-carboxylate at 10.0 mg/kg feed; LO, indole-3-carboxylate at 1.0 mg/kg feed. All chickens, except for CON, were infected by oral gavage at day 14 with 1.0 × 10^4^ oocysts/chicken of *E. maxima*. ^a~c^ Bars with no common letter differ significantly (*p* < 0.05). Each bar represents the mean ± SEM (n = 6). The data were collected on day 20 (6 days post-infection). Transcript levels of the tight junction proteins were measured using quantitative RT-PCR and normalized to GAPDH transcript levels.

The gene expression of jejunal claudin-2 (*p* = 0.001, 7.4 × 10^−2^ to 4.1 × 10^−2^) and ZO-2 (*p* < 0.001, 2.5 × 10^−1^ to 1.2 × 10^−1^) was decreased by *E. maxima* infection (NC) compared to that of uninfected group (CON), whereas HI increased the claudin-2 (*p* = 0.043, 4.1 × 10^−2^ to 5.9 × 10^−2^) and ZO-2 (*p* = 0.048, 1.2 × 10^−1^ to 1.8 × 10^−1^) levels compared to that of NC (**Figures 8B, F**). The gene expression of jejunal JAM-2 in the LO group was increased (*p* < 0.035, 4.8 × 10^−3^ to 1.0 × 10^−3^) compared to that of the other groups (**Figure 8C**). In the case of occludin, the HI (*p* = 0.010, 1.4 × 10^−2^ to 2.5 × 10^−2^) and LO (*p* = 0.010, 1.4 × 10^−2^ to 3.3 × 10^−2^) groups increased the occludin levels compared to those of CON (**Figure 8D**). However, claudin-1 (**Figure 8A**) and ZO-1 (**Figure 8E**) levels were not changed by *E. maxima* infection or ICOOH administration.

#### 3.2.4 Jejunal Lesion Scores and Fecal Oocyst Shedding

**Figure 9:**
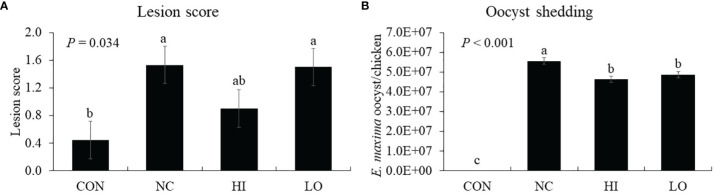
Lesion score and oocyst shedding of chickens fed diet supplemented with indole-3-carboxylate during infection with *Eimeria maxima*. CON, basal diet; NC, basal diet for infected chickens; HI, indole-3-carboxylate at 10.0 mg/kg feed; LO, indole-3-carboxylate at 1.0 mg/kg feed. All chickens, except for CON, were infected by oral gavage at day 14 with 1.0 × 10^4^ oocysts/chicken of *E. maxima*. ^a~c^Bars with no common letter differ significantly (*p* < 0.05). Each bar represents the mean ± SEM (n = 6). The lesion score was collected from distal jejunal tissue on day 20 [6 days post-infection (dpi)], and fecal sample was collected from 6 to 8 dpi to calculate the oocyst shedding.

Chickens infected with *E. maxima* (NC) showed jejunal lesion scores (0.4 to 1.5), whereas none of the uninfected CON group showed any lesion (**Figure 9A**). The jejunal lesion score of the HI group did not differ (*p* = 0.254) from that of the NC group statistically even though the values for the HI group decreased (0.4 to 0.9) numerically by 50%. Within the infected groups, all groups demonstrated increased (*p* < 0.001) oocyst numbers (0 to 5.0 × 10^7^ oocysts/chicken) compared to those of the CON group. Among these groups, the HI (*p* = 0.004, 5.6 × 10^7^ to 4.6 × 10^7^ oocysts/chicken) and LO (*p* = 0.017, 5.6 × 10^7^ to 4.9 × 10^7^ oocysts/chicken) groups decreased the fecal oocyst number compared to that of the NC group (**Figure 9B**).

#### 3.2.5 Nutrient Transporters

The infection with *E. maxima* (NC) decreased the gene expressions of jejunal CAT1 (*p* = 0.001, 0.055 to 0.016), EAAT (*p* < 0.001, 0.12 to 0.040), GLUT2 (*p* = 0.009, 0.036 to 0.008), GLUT5 (*p* = 0.001, 0.24 to 0.096), and LAT1 (*p* < 0.001, 0.092 to 0.018) compared to those of CON, whereas HI increased the gene expressions of jejunal CAT1 (*p* = 0.041, 0.016 to 0.037), EAAT (*p* = 0.006, 0.040 to 0.077), GLUT2 (*p* = 0.017, 0.008 to 0.033), GLUT5 (*p* = 0.025, 0.096 to 0.185), and LAT1 (*p* = 0.028, 0.018 to 0.055) compared to those of NC (**Table 3**). Jejunal CAT1 (*p* = 0.033, 0.016 to 0.038) and EAAT (*p* = 0.034, 0.040 to 0.066) levels were also increased in the LO group compared to those of NC. However, jejunal B^0^AT, B^0+^AT, GLUT1, LAT2, and SGLT levels were not changed (*p* > 0.05) by infection or dietary ICOOH supplementation.

**Table 3:** Jejunal nutrient transporters of chicken fed a diet supplemented with indole-3-carboxylate.

Item	CON	NC	HI	LO	SEM	*p*-Value
Mean normalized expression^1)^						
B^0^AT	0.38	0.14	0.27	0.18	0.059	0.063
B^0+^AT	0.070	0.031	0.055	0.074	0.011	0.057
CAT1	0.055^a^	0.016^b^	0.037^a^	0.038^a^	0.007	0.007
EAAT	0.12^a^	0.040^c^	0.077^b^	0.066^b^	0.008	< 0.001
GLUT1	0.0023	0.0017	0.0022	0.0027	0.0005	0.496
GLUT2	0.036^a^	0.0084^b^	0.0327^a^	0.0180^ab^	0.006	0.028
GLUT5	0.24^a^	0.096^c^	0.185^b^	0.0987^c^	0.025	0.002
LAT1	0.092^a^	0.018^c^	0.055^b^	0.049^bc^	0.011	0.002
LAT2	0.0009	0.0012	0.0011	0.0012	0.0004	0.858
SGLT	0.34	0.23	0.30	0.20	0.045	0.152

SEM (n = 6/treatment).

CON, basal diet; NC, basal diet for Eimeria maxima-infected chickens; HI, indole-3-carboxylate at 10.0 mg/kg feed; LO, indole-3-carboxylate at 1.0 mg/kg feed; B^0^AT, Na+-dependent amino acid transporter; B^0+^AT, Na+-independent amino acid transporter; CAT1, cationic amino acid transporter; EAAT, excitatory amino acid transporter; GLUT1, glucose transporter 1; GLUT2, glucose transporter 2; GLUT5, glucose transporter 5; LAT1, L-type amino acid transporter 1; LAT2, Na^+^-dependent neutral/cationic amino acid transporter; SGLT, sodium-glucose transporter.

^1^Transcript levels of the nutrient transporters were measured using quantitative RT-PCR and normalized to GAPDH transcript levels.

^a~c^Means in the same row with different superscripts differ (p < 0.05), and the difference was re-evaluated by PDIFF option in SAS when p-value between treatments was less than 0.05.

#### 3.2.6 Growth Performance of Chickens

The initial BW did not exhibit remarkable differences among the experimental groups (*p* = 1.00; **Table 4**). Until day 14 without the development of infection, treatment with dietary ICOOH supplementation did not significantly change the BW of chickens compared to that of the control group (CON and NC). *E. maxima* infection (NC) decreased (*p* < 0.001) the BW of chickens at 6 dpi (861 to 742 g) and 8 dpi (1,018 to 761 g) compared to that of the CON group. There was no difference (*p* > 0.05) between infected groups at 6 dpi. The LO group increased (*p* = 0.011) the BW (761 to 850 g) of chickens at 8 dpi compared to that of the NC group. However, the HI group did not change the BW of chickens at 8 dpi compared to that of the NC group even though the HI group increased the BW (761 to 809 g) of chickens numerically compared to that of the NC group. In the case of ADG, before infection, the ADG of the chickens fed with diets supplemented with ICOOH did not differ (*p* > 0.05) from that of the control groups (CON and NC). *E. maxima*-infected chickens (NC group) presented (*p* < 0.001) with lower ADG (66.5 to 47.2 g) at 0 to 6 dpi compared to that of the uninfected CON group. However, HI and LO groups did not change the ADG of chickens from 0 to 6 dpi. Like the BW, the LO group significantly increased (*p* = 0.005) the ADG (38.0 to 47.0 g) of the infected chickens at 0 to 8 dpi. Nevertheless, the HI group increased the ADG (38.0 to 43.6 g) of chickens only numerically compared to that in the NC group.

**Table 4:** Body weight and average daily gain chickens fed a diet supplemented with indole-3-carboxylate.

Treatment	CON	NC	HI	LO	SEM	*p*-Value
Body weight, g						
Initial	36.6	36.6	36.6	36.6	0.7	1.000
D 7	155	156	156	157	3.0	0.900
D 14 (0 dpi)	460	458	461	468	7.8	0.803
D 20 (6 dpi)	861^a^	742^b^	743^b^	756^b^	19	0.001
D 22 (8 dpi)	1,018^a^	761^c^	809^b^	850^b^	23	< 0.001
Average daily gain, g						
D 0 to 7	16.9	17.0	17.1	17.2	0.4	0.607
D 7 to 14	43.5	43.1	43.5	44.2	0.8	0.271
D 0 to 14^1)^	32.7	32.2	32.6	32.8	0.6	0.894
D 14 to 20	66.5^a^	47.2^b^	47.1^b^	47.7^b^	2.3	< 0.001
D 14 to 22^2)^	69.7^a^	38.0^c^	43.6^bc^	47.0^b^	2.1	< 0.001

All chickens, except for CON, were infected by oral gavage at day 14 with 1.0 × 10^4^ oocysts/chicken of Eimeria maxima. SEM (5 chickens/cage and 6 cages/treatment until day 20 and 4 chickens/cage and 6 cages/treatment after day 20), ^1^before infection and ^2^after infection.

CON, basal diet; NC, basal diet for E. maxima-infected chickens; HI, indole-3-carboxylate at 10.0 mg/kg feed; LO, indole-3-carboxylate at 1.0 mg/kg feed; ADG, average daily gain; BW, body weight; D, day; dpi, days post-infection.

^a~d^Means in the same row with different superscripts differ (p < 0.05), and the difference was re-evaluated by PDIFF option in SAS when p-value between treatments was less than 0.05.

### 4 Discussion

Epithelial TJs are the key structures regulating paracellular space and the passage of macromolecules and microorganisms. Thus, controlling the barrier integrity is important in preventing enteric inflammation (29). The TJs are composed of multiprotein complexes in charge of controlling selective permeability through sealing between adjacent epithelial cells, which are located at the apical ends of the lateral membranes of IECs (30, 31). Permeation of intestinal pathogens caused by disruption of the intestinal TJ barrier can act as a trigger for inflammation and host immune responses, which may follow the development of local (intestine) and systemic diseases (32). In this paper, *in vitro* tests were used to measure gene changes in occludin and ZO-1 as TJ proteins in IECs as well as MUC-2, mucin protein. Al-Sadi et al. (33) demonstrated that occludin plays a role in the maintenance and assembly of TJ proteins through an experiment that increased paracellular permeability to macromolecules by using knockdown of occludin in Caco-2 cells and mouse intestine. In the case of ZO-1, ZO-1 deficiency did not change TJ structures or permeability; however, suspended assembly of occludin and claudin was observed (34). Thus, ZO-1 may also be associated with the TJ regulation. MUC-2 gene deficiency of mice caused changes in biochemistry and molecular pathways; thereby the probability of tumor was facilitated (35). In the current study, ICOOH at 10.0 µg/ml increased gene expressions of occludin, ZO-1, and MUC-2 in IECs compared to those of ICOOH at 0.0, 0.1, and 1.0 µg/ml. Other indole compounds have also shown improvements in epithelial barrier function. Three tryptophan metabolites (indole-3-ethanol at 600 mg/kg mice, indole-3-pyruvate at 2,900 mg/kg mice, and indole-3-aldehyde at 1,000 mg/kg mice) administration increased gene expression levels of occludin and ZO-1, thereby enhancing adhesive forces between adjacent epithelial cells in order to seal the weakened intestinal barrier of colitis mice induced by dextran sodium sulfate (36). Indole-3-propionic acid (I3P) administration in the Caco-2/HT29 coculture model improved occludin, ZO-1, and MUC-2 (37), as well as reduced the expression of LPS-induced inflammatory factors. This supports our results that ICOOH reduced the gene expression levels of IL-1β and IL-8, which were increased by LPS stimulation in CMCs. Recently, there has been increasing evidence that cytokines and TJ proteins have a co-dependent relationship to maintain intestinal homeostasis against unbalanced physiological conditions in the intestine due to host immune responses, inflammation, or invasion of pathogens (29, 38, 39). Interleukins are a large family of cytokines, and several ILs such as IL-1, 2, 4, 6, 8, and 10 have been researched for their effects on paracellular permeability (38). In the case of IL-1β and IL-8, which are markedly expressed in intestinal mucosa under inflammatory conditions, there was a report that the release of IL-1β and IL-8 increased permeability between intestinal barriers due to weakened TJ proteins (40, 41). Based on the result of ICOOH administration as shown in IEC and CMC cultures, ICOOH improved TJ protein expression as well as regulated cytokines.

Interestingly, an indole-based scaffold (indole-3,3′-indolizoidine), which was found from natural products, reduced *Plasmodium* (malaria parasite) *via* a molecular mechanism of ionic imbalance mediated cell death (42). Since both *Eimeria* and *Plasmodia* belong to the Apicomplexa phylum, ICOOH may have a similar function to inhibit the growth of these protozoan parasites. Other indole compounds are antiplasmodial agents. For example, aminoindole (43), bisindole (44), piperidine indole (45), or spiroindolone (46) are known as scaffolds in the novel chemical classes of antiplasmodial agents exhibiting various modes of action, of which, aiming at the formation of hemozoin in *Plasmodium falciparum*, PfATP4 and melatonin receptor are the most commonly recognized modes of actions (47). Therefore, we evaluated whether ICOOH has any direct effect on sporozoite of *E. maxima*. Chicken NK-lysin-derived peptide (cNK-lysin), which has an anti-coccidia function, was used as a positive control (23), and these cNK-lysins attenuated their survival ratios by 48% to 12% dose-dependently. However, ICOOH did not show any effect on the survival ratios of the sporozoite. Thus, ICOOH does not have any direct effect on the sporozoite of *E. maxima*.

Indole-6-carboxaldehyde was shown to prevent C2C12 myoblast DNA damage and apoptosis caused by oxidative stress, and the mechanism was associated with the ROS-AMPK signaling pathway (48). Also, dietary I3P as a metabolite produced by gut microbiota changed the BW of rats (49). We therefore evaluated the effects of ICOOH on muscle cell growth by using QMCs and PMCs *in vitro*. In the present study, we used different FBS concentrations (0.5% FBS in QMCs and 2% FBS in PMCs) to induce myogenic differentiation as in previous studies (50). In the current study, the MyoG level, when using the lowered FBS concentration, was elevated, thus supporting MyoG as a differentiation marker. During myogenic differentiation induced by the lowered FBS concentrations, ICOOH administration at a dose of 0.1 µg/ml enhanced MyoG levels in QMCs, but it did not change in PMCs. QMCs and PMCs presently have different cell growth properties, as PMCs are undifferentiated primary cells obtained from chicken embryos, whereas QMCs are established muscle cells obtained from quails. Lower FBS concentrations or ICOOH administration did not affect Pax7 levels in QMCs and PMCs.

To study the role of ICOOH in chickens, the effects of dietary ICOOH at high and low doses on intestinal immune responses, epithelial barrier integrity, and growth performance of chickens following an *E. maxima* challenge were examined.

Induction of intestinal cytokine gene expression during *Eimeria* infection in the gut in chickens depends on various conditions, including *Eimeria* spp., time after infection, ingested *Eimeria* dose, infection site, and infection methods (51–53). In our current study, *E. maxima* infection at 6 dpi elevated a vast majority of jejunal cytokine levels, which were related to inflammation and Th cell stimulation. According to previous studies, intestinal cytokine genes are normally expressed from 2 dpi, peaking at 6 or 7 dpi following coccidiosis, but their expression is reduced dramatically after the peak. In the case of proinflammatory cytokines such as TNFSF15, IL-1β, IL-6, and IL-8, dietary ICOOH supplementation suppressed the gene expression of jejunal IL-1β of *E. maxima*-infected chickens in both HI and LO. Additionally, jejunal IFN-γ and IL-10, which play a role in the regulation of Th cells, were also suppressed by dietary ICOOH supplementation. Based on these results, dietary ICOOH supplementation may affect not only the regulation of cytokines related to inflammation responses in the jejunum of chicken infected with *E. maxima* but also control Th cell responses through regulating cytokines related to Th cells. The major roles of IL-1β are executing coordination between host immune and proinflammatory responses and enhancing the production of cytokines and chemokines (54, 55). Therefore, the interaction of IL-1β and its receptor facilitates a cascade of immune responses (56). Moreover, IFN-γ is also known as a major cytokine mediating resistance to the challenge of *Eimeria* spp. or *Salmonella* spp (53).. Increased IFN-γ causes recruitment and stimulation of CD4^+^ and TCR2^+^ cells (57). In the case of IL-10, which is mainly produced by activated macrophages or CD4^+^ and CD8^+^ T cells, it executes the regulation of innate and cell-mediated immune responses simultaneously or independently (58, 59).

Nevertheless, in terms of poultry nutrition, releasing various cytokines can be considered as the diversion of nutrients to produce various immune-related proteins, which may cause a waste of valuable nutrition for chicken growth (60). Thus, we speculated that reduced IL-1β, IFN-γ, and IL-10 by dietary ICOOH supplementation may not only show regulation of inflammation responses and Th cells but also lead to the supply of dietary nutrients properly in harmony with the fundamental purpose of feed. Additionally, our results are supported by other studies reporting the immunoregulatory properties of indole compounds on innate immunity and T cells. For example, Whitfield-Cargile et al. (61) have asserted that dietary indole attenuated multiple deleterious effects of mucosal inflammation caused by non-steroidal anti-inflammatory drugs, indomethacin, in mice.

Kim et al. (19) have also reported that dietary 3,3′-diindolymethane and indole-3-carbinol supplementation alleviates intestinal tissue damage of chicken infected by *Eimeria tenella*. In that study, dietary indoles carried out immune-modulating property on T-cell immunity through activation of intestinal (AhR). Thus, in the current study, CYP1A4 and CYP1A5, enzymes related to activation of the AhR as cytochrome P450 family members in chickens (62, 63), were measured to obtain a magnitude of AhR activation. Dietary ICOOH supplementation improved these gene expressions of jejunal CYP1A4 and CYP1A5 compared to that of *E. maxima* infected chicken. Based on these results, we speculated that dietary ICOOH may also work through intestinal AhR activation, along with 3,3′-diindolymethane and indole-3-carbinol. Many studies have demonstrated that AhR activation is associated with the regulation of various cellular functions related to antitumor and antioxidant activities and immunoregulatory properties since AhR was observed as a transcription factor mediating the toxicity of chemicals such as 2,3,7,8-tetrachlorodibenzo-*p*-dioxin (64–66). Early studies on the AhR activation were focused on its ability to sense tumorigenesis of the colon through mice lacking the AhR pathway. However, the sensing ability affects colon cancer and also protects against inflammatory damage by maintaining intestinal cell homeostasis and barrier integrity (67). Especially, findings (68) of an evolutionarily conserved binding site for AhR in *foxp3* gene and three non-evolutionarily conserved AhR-binding sites in promoter regions of *foxp3* genes have led to observations that AhR can induce the generation of Treg cells through controlling *foxp3* expression. In addition to the role of *foxp3* gene, several microRNAs, which are targeting *foxp3* and IL-17 mRNA, affect functional activities of T-cell responses through Treg cell induction and suppression of Th2 and Th17 cells according to the type of AhR ligands (69). Unfortunately, because a foxp*3* homolog in poultry has not been identified yet, it is not possible to know the role of AhR in *foxp3* activation (19). Schiering et al. (66) established an important role of the AhR pathway in IECs as a “gatekeeper” for scanning proper ligands of the entire immune cells in the intestine (not confining the role of the AhR in only T-cell responses). Moreover, Metidji et al. (67) found more IEC-intrinsic AhR functions that maintain intestinal cell homeostasis and barrier integrity to protect against inflammatory damage. In the current study, we provided the first demonstration of how dietary ICOOH as AhR ligand modulates jejunal AhR activation in chicken through gene expression of CYP1A4 and CYP1A5.

Dietary ICOOH modulated the expression of TJ proteins to enhance barrier integrity following *E. maxima* infection. *E. maxima* infection reduced gene expression of jejunal claudin-2 and ZO-2 by approximately 2-fold compared to that of the CON group, whereas dietary ICOOH at 10.0 mg/kg feed improved them compared to the NC group. *E. maxima* challenge did not change the gene expression of other TJ proteins such as claudin-1, JAM-2, occluding, and ZO-1, most likely due to the fact that the challenge resulted in mild coccidiosis as determined by a lesion score of only 1.5. ICOOH at 1.0 mg/kg improved gene expression of jejunal JAM-2 and occludin regardless of *E. maxima* infection. Dietary indole supplementation has been shown to enhance gene expression of cecal JAM-2 and ZO-1 of chicken infected with *E. tenella* (19), consistent with our finding that dietary indole supplementation affects TJ proteins to enhance intestinal barrier integrity of chicken infected with *E. maxima*. Shimada et al. (70) also reported the effect of indole on enhanced epithelial barrier function in the colon of germ-free (GF) and specific pathogen-free mice. In the study, dietary indole was demonstrated to improve cecal JT proteins of dextran sulfate sodium (DSS)-induced GF mice. The role of indole as a quorum-sensing molecule may be implicated in binding to specific receptors and stimulating a signaling pathway related to the regulation of host IECs (71).

In this study, we also investigated the role of various nutrient transporters following dietary ICOOH in *E. maxima* infection. A total of 10 nutrient transporters were measured, and 5 (CAT1, EAAT, GLUT2, GLUT5, and LAT1) of them were significantly changed by *E. maxima* infection and dietary ICOOH supplementation. They showed regular patterns, which were mainly reduced by infection and were improved by ICOOH at 10.0 mg; however, the other 5 examined were unaffected. The major role of the small intestine, especially jejunum, in chicken, is absorbing many nutrients from feed, and the nutrient transporters, which are located at the brush border membrane of intestinal cells, mediate the nutrient absorption (72). In the current study, *E. maxima* infection may cause jejunal destruction but as mentioned above, the magnitude of the impact of *E. maxima* infection, confirmed through lesion score in this study, was mild. Due to the mild condition, we speculated that some transporters may be unaffected. In the current study, these transporters improved by dietary ICOOH supplementation may help the absorption of more nutrients, which may be utilized for overcoming the *E. maxima* challenge.

Chickens that were treated with ICOOH showed reduced oocyst shedding by 18% in the HI group and 12% in LO compared to that of the infected group (NC). As a direct effect on *Eimeria* was not found in the *in vitro* results and given that dietary ICOOH improved jejunal nutrient transporters as well as immune responses (especially inflammatory and T-cell responses), TJ proteins, and AhR in the current study, reduction of parasite fecundity may be affected by improved host immunity.

Chicken on the LO diet had improved BW at 8 dpi compared to that of NC. We speculate that the growth-promoting effect of chickens on dietary ICOOH may be affected by the beneficial effects of the suppressed cytokines, the activated AhR, the increased TJ proteins and nutrient transporters, and the reduced oocyst shedding. In conclusion, indole-3-carboxylate enhanced the expression of TJ proteins, reduced the expression of proinflammatory cytokines *in vitro*, and exerted beneficial effects by reducing intestinal damage, parasite fecundity, and inflammatory responses in broilers in an *E. maxima* challenge model. This corroborates previous data indicating that indole metabolites were increased in the gut of young broiler chickens, which had improved immunity and growth, due to a dietary feed additive containing *B. subtilis* strains 1781 and 747. Therefore, indole-3-carboxylate is a potential postbiotic candidate to improve the growth and immunity of chickens presenting with enteric diseases.

## Data Availability Statement

The original contributions presented in the study are included in the article/supplementary material. Further inquiries can be directed to the corresponding author.

## Ethics Statement

The animal study was reviewed and approved by The Beltsville Agricultural Research Center Institutional Animal Care and Use Committee.

## Author Contributions

IP and HL designed the research. HL supervised the research. IP, DG, and HN conducted the *in vitro* research. IP and SW conducted the *in vivo* research. IP analyzed the data and drafted the manuscript. HL supervised the research and edited the manuscript. IP, HN, DG, SW, NZ, AS, TR, and HL had responsibility for the content. All authors listed have made a substantial, direct, and intellectual contribution to the work and approved it for publication.

## Funding

Funding was partly supported by ARS CRIS 8042-32000-115-00D.

## Conflict of Interest

Authors NZ, AS, and TR were employed by the Arm & Hammer Animal and Food Production.

The remaining authors declare that the research was conducted in the absence of any commercial or financial relationships that could be construed as a potential conflict of interest.

## Publisher’s Note

All claims expressed in this article are solely those of the authors and do not necessarily represent those of their affiliated organizations, or those of the publisher, the editors and the reviewers. Any product that may be evaluated in this article, or claim that may be made by its manufacturer, is not guaranteed or endorsed by the publisher.
